# Supplementary material for: Autocrine Bradykinin Release Promotes Ischemic Preconditioning-Induced Cytoprotection in Bovine Aortic Endothelial Cells
Source: Int J Mol Sci. 2020 Apr 23;21(8):2965. doi: 10.3390/ijms21082965 (PMC7215376; doi:10.3390/ijms21082965)
Supplement: Supplementary file 1 [file ijms-21-02965-s001.pdf]

SUPPLEMENTARY MATERIAL

Autocrine Bradykinin Release Promotes Ischemic Preconditioning-Induced Cytoprotection in Bovine Aortic Endothelial Cells.

Alessandro Bellis [1,2], Daniela Sorriento [1], Antonella Fiordelisi [1], Raffaele Izzo [1], Junichi Sadoshima [3], Ciro Mauro [2], Federica Cerasuolo [1], Costantino Mancusi [1], Emanuele Barbato [1], Emanuele Pilato [4], Bruno Trimarco [1] and Carmine Morisco [1,*]

[1] Dipartimento di Scienze Biomediche Avanzate, Università FEDERICO II, via S. Pansini n. 5, 80131, Napoli; abellis82@vodafone.it (A.B.); daniela.sorriento@unina.it (D.S.); antonella.fiordelisi@gmail.com (A.F.); rafizzo@unina.it (R.I.); f.andrea_cerasuolo@hotmail.it (F.C.); costantino.mancusi@unina.it (C.M.); emanuele.barbato@unina.it (E.B.); trimarco@unina.it (B.T.)

[2] Dipartimento Emergenza Accettazione, Unità Operativa Complessa Cardiologia con UTIC ed Emodinamica, Azienda Ospedaliera "Antonio Cardarelli", via A. Cardarelli n. 9, 80131, Napoli; ciro.mauro1957@gmail.com (C. M.)

[3] Department of Cell Biology and Molecular Medicine, Rutgers–New Jersey Medical School, 185 S. Orange Ave, MSB G609, Newark, NJ 07103; sadoshju@njms.rutgers.edu (J. S.)

[4] Dipartimento di Emergenze Cardiovascolari, Medicina Clinica e dell'Invecchiamento, Azienda Ospedaliera Università, FEDERICO II, via S. Pansini n. 5, 80131, Napoli; emapilato@yahoo.it

* Correspondence: carmine.morisco@unina.it (C. M.); Tel.: +39-081-7462253; Fax: +39-081-7462256

Results

PC Does Not Increase Tissue Kallikrein (KLK1) Protein Expression in bAECs

Since we have demonstrated that ischemic preconditioning (PC) does not increase mRNA levels of KLK1 (Figure 1 C in the manuscript text), we also assessed protein expression of KLK1 in bAECs exposed to the PC stimulus for the indicated durations. Consistently with mRNA levels, PC does neither increase protein expression of KLK1 (Figure S1).

Therefore, bradykinin (Bk) synthesis in the two phases of PC cannot be attributed to augmented expression of this protein.

PC-Induced Bk Synthesis Reduces Caspase-3 Cleavage in bAECs Exposed To Prolonged Hypoxia

Since caspase-3 plays a key role in regulation of the cellular suicide cascade [1], we assessed caspase-3 cleavage in early and late preconditioned bAECs exposed to prolonged hypoxia in the presence or absence of aprotinin (AP, 5 µg). AP pretreatment increased caspease-3 cleavage in early and late preconditioned cells compared to in non-pretreated early and late preconditioned cells (Figure S2 and Table S1). Consistently, stimulation of bAECs with concentrations of exogenous Bk comparable to those found in culture media from early and late preconditioned cells (10^{-12} M and 10^{-11} M) decreased caspase-3 cleavage compared to in non-preconditioned cells (Figure S2 and Table S1).

These data indicate that, in endothelial cells, PC-mediated Bk release through an autocrine mechanism accounts for cytoprotection against prolonged hypoxia-induced apoptosis.

PC Reduces Caspase-3 Cleavage in bAECs Exposed To Prolonged Hypoxia Through Bk Receptors (BKRs) Activation

We assessed caspase-3 cleavage in early and late preconditioned bAECs exposed to prolonged hypoxia in the presence or absence of selective BKRs inhibitors. Cell pretreatment with HOE-140 (a selective BKR2 inhibitor) increased caspase-3 cleavage in both PC phases (Figure S3 B; in contrast, pretreatment with desArg10-HOE-140 (a selective BKR1 inhibitor) only did in late PC (Figure S3 A and Table S2).

These results suggest that BKR2 plays a predominant role in PC-mediated caspase-3 cleavage decrease, which is subsequently supported by increased expression of BKR1 in late PC.

Transient Transfection of Selective siRNAs for Bk Receptor 1 and 2 Abrogates PC-Induced Cytoprotection

In order to confirm the inhibition of PC-induced cytoprotection obtained with chemical inhibitors of BKRs 1 and 2 (desArg10-HOE-140 and HOE-140, respectively), we silenced BKRs expression by transient transfection of bAECs with specific siRNAs. In particular, we assessed cell death by AV/PI staining in early and late preconditioned bAECs exposed to a prolonged hypoxia (12 hours), in absence and in presence of these selective siRNAs for BKRs 1 and 2 (20 g). Similarly to pretreatment with receptor antagonists, BKRs2 silencing abolished anti-apoptotic effect in both phases of PC, whereas BKRs1 silencing did only in late PC (Figure S4 A and Table S2). These results were confirmed by assessment of caspase-3 cleavage (Figure S4 B).

These findings confirm the preminent role of BKRs2 in induction of PC-mediated cytoprotection.

Blocking PC-Mediated Endocytosis and Recycling of BKR2 Increases Caspase-3 Cleavage

We assessed caspase-3 cleavage in early and late preconditioned bAECs exposed to prolonged hypoxia in the presence or absence of the endocytosis inhibitor monodansylcadaverine and of the recycling inhibitor bafilomycin. Cell pretreatment with monodansylcadaverine and bafilomycin increased caspase-3 cleavage in both PC phases (Figure S5 and Table S2).

These findings indicate that recycling and endocytosis of BKR2 are required for the PC-induced caspase-3 cleavage decrease.

Aprotinin Pretreatment Abrogates PC-Dependent Akt Phosphorylation

To further underline the key role of kinin-kallikrein system in PC phenomenon, we assessed Akt phosphorylation in serine473 residues during early and late PC, in presence and absence of aprotinin (AP, 5 µg), a selective KLK1 inhibitor. AP abrogated Akt phosphorylation in both phases of PC (Figure S6), demonstrating that this enzyme, deputed to Bk synthesis, is required to induce activation of this kinase implied in protective molecular pathways of bAECs.

Supplemental Figures

Figure S1

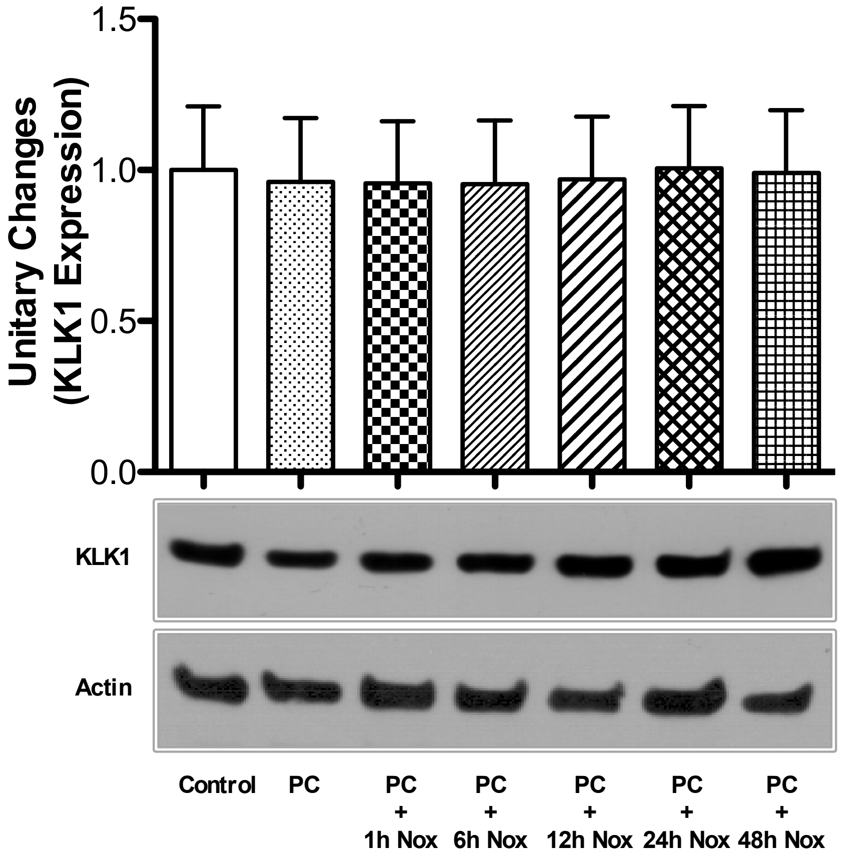

Figure 1. Cells were subjected to preconditioning (PC). Tissue Kallikrein (KLK1) protein expression was assessed at different times following PC (Nox: normoxia). Bar graph represents densitometric analysis, mean±SEM, expressed as fold increase in caspase-3 cleavage over that in control cells, of three independent experiments.

Figure S2

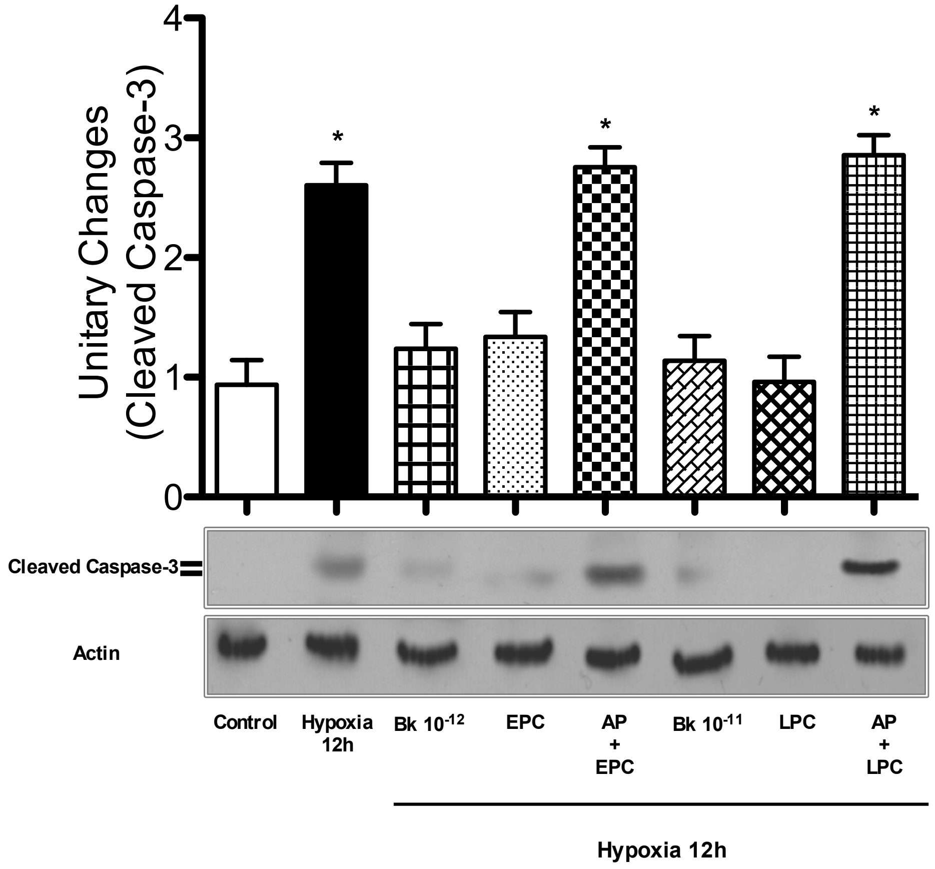

Figure 2. Cells were subjected to early and late preconditioning (EPC and LPC), in absence and in presence of aprotinin (AP), as reported in the text. Consistently, cells were stimulated with concentrations of exogenous bradykinin (Bk) comparable to those found in culture media from early and late preconditioned cells (10^{-12} M and 10^{-11} M). Caspase-3 cleavage was assessed. Bar graph represents densitometric analysis, mean±SEM, expressed as fold increase in caspase-3 cleavage over that in control cells, of three independent experiments.* $p<0.001$ vs control, by one-way ANOVA with a post hoc test of HSD.

Figure S3

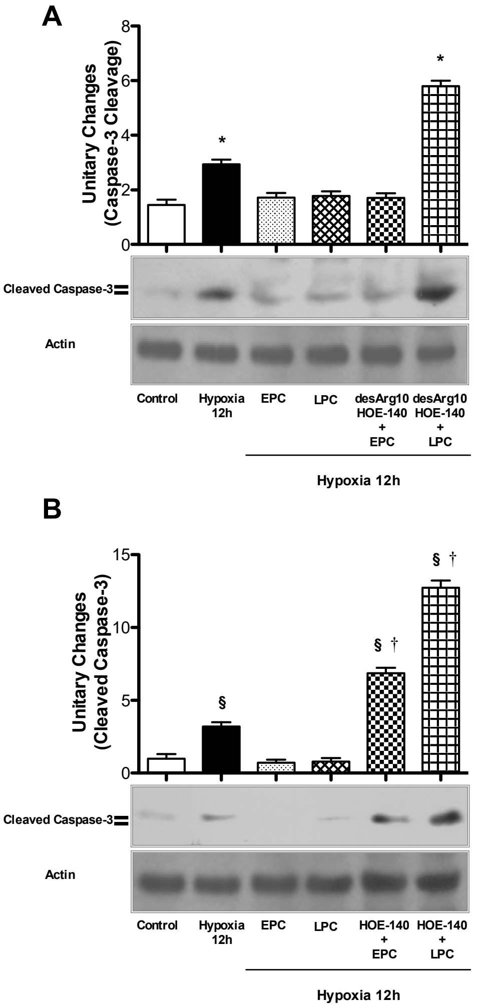

Figure 3. Cells were subjected to early and late preconditioning (EPC and LPC), in absence and in presence of (A) selective Bk receptor 1 (BKR1) inhibitor desArg10-HOE-140 and of (B) selective Bk receptor 2 (BKR1) inhibitor HOE-140, as reported in the text. Caspase-3 cleavage was assessed. Bar graph represents densitometric analysis, mean±SEM, expressed as fold increase in caspase-3 cleavage over that in control cells, of four independent experiments.* $p<0.001$ vs control, EPC, LPC and desArg10-HOE-140+EPC; § $p<0.001$ vs control, EPC and LPC; † $p<0.001$ vs hypoxia 12 h, by one-way ANOVA with a post hoc test of HSD.

Figure S4

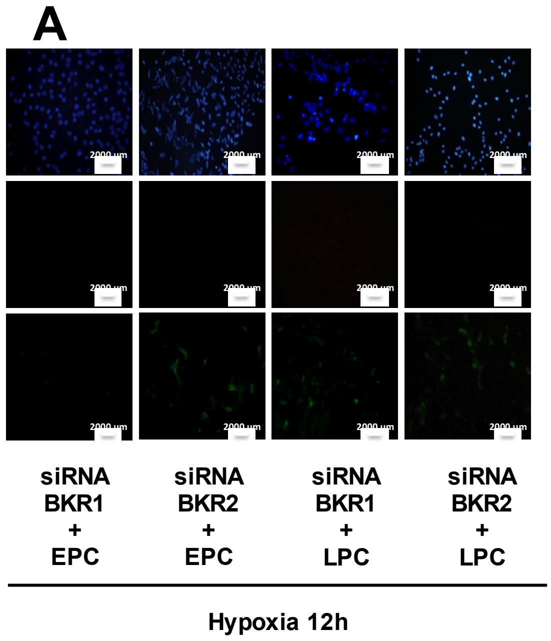
A
siRNA BKR1 + EPC
siRNA BKR2 + EPC
siRNA BKR1 + LPC
siRNA BKR2 + LPC
Hypoxia 12h

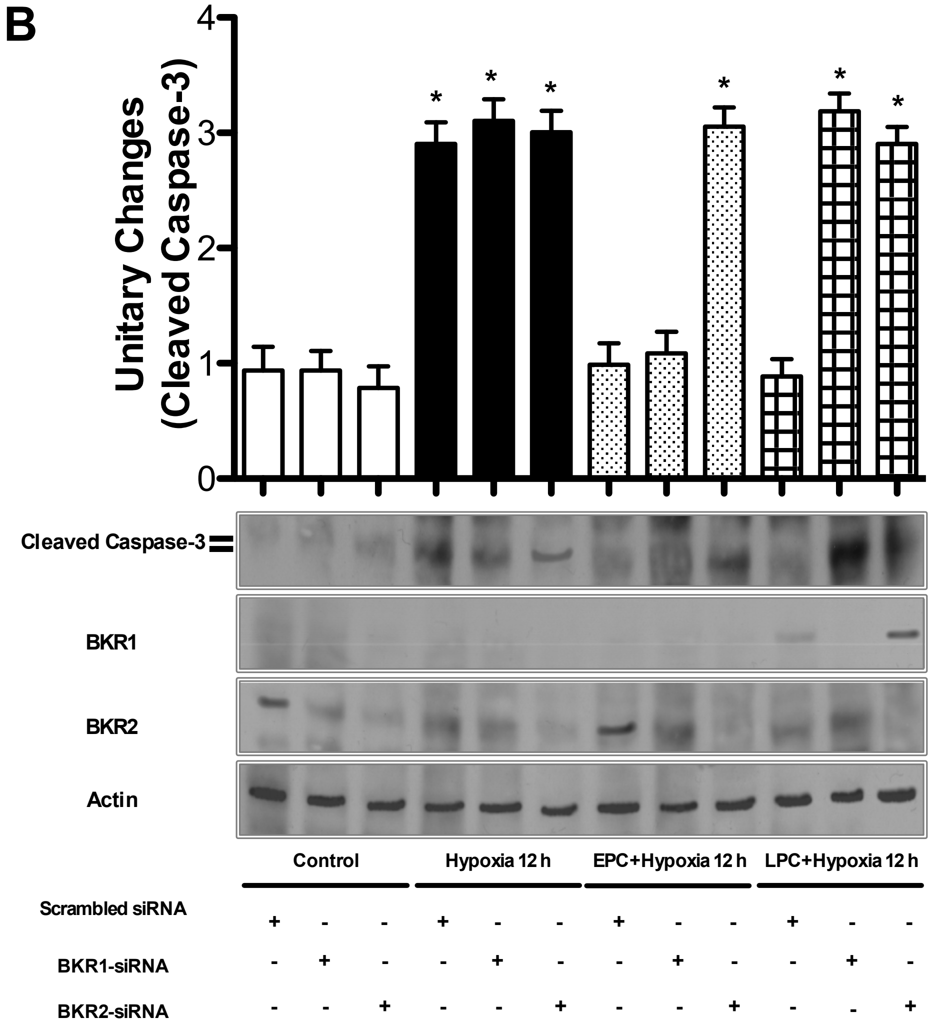
B
Unitary Changes (Cleaved Caspase-3)
0
1
2
3
4
Cleaved Caspase-3
BKR1
BKR2
Actin
Control
Hypoxia 12 h
EPC+Hypoxia 12 h
LPC+Hypoxia 12 h
Scrambled siRNA
BKR1-siRNA
BKR2-siRNA

Figure 4. Cells were transfected with BKR2- and BKR1- short interfering RNA (BKR2- and BKR1-siRNA), then subjected to early and late PC. (A) Apoptosis was assessed by Annexin V (green) and necrosis by Propidium Iodide (red) staining, and nuclei were stained with DAPI (blue). The rates of apoptosis and necrosis were calculated by dividing the number of Annexin-V-positive/PI-negative cells and Annexin-V-positive/PI-positive cells respectively by the total number of nuclei detected with DAPI staining. The percentage of apoptotic and necrotic cell death was calculated from six independent experiments. Quantification of apoptosis and necrosis is reported in Table II of supplement material. The size of the scale bar is 2000 m. (B) Caspase-3 cleavage was also assessed. Bar graph represents densitometric analysis, mean±SEM, expressed as fold increase in caspase-3 cleavage over that in control cells, of three independent experiments.* $p<0.001$ vs control, by one-way ANOVA with a post hoc test of HSD.

Figure S5

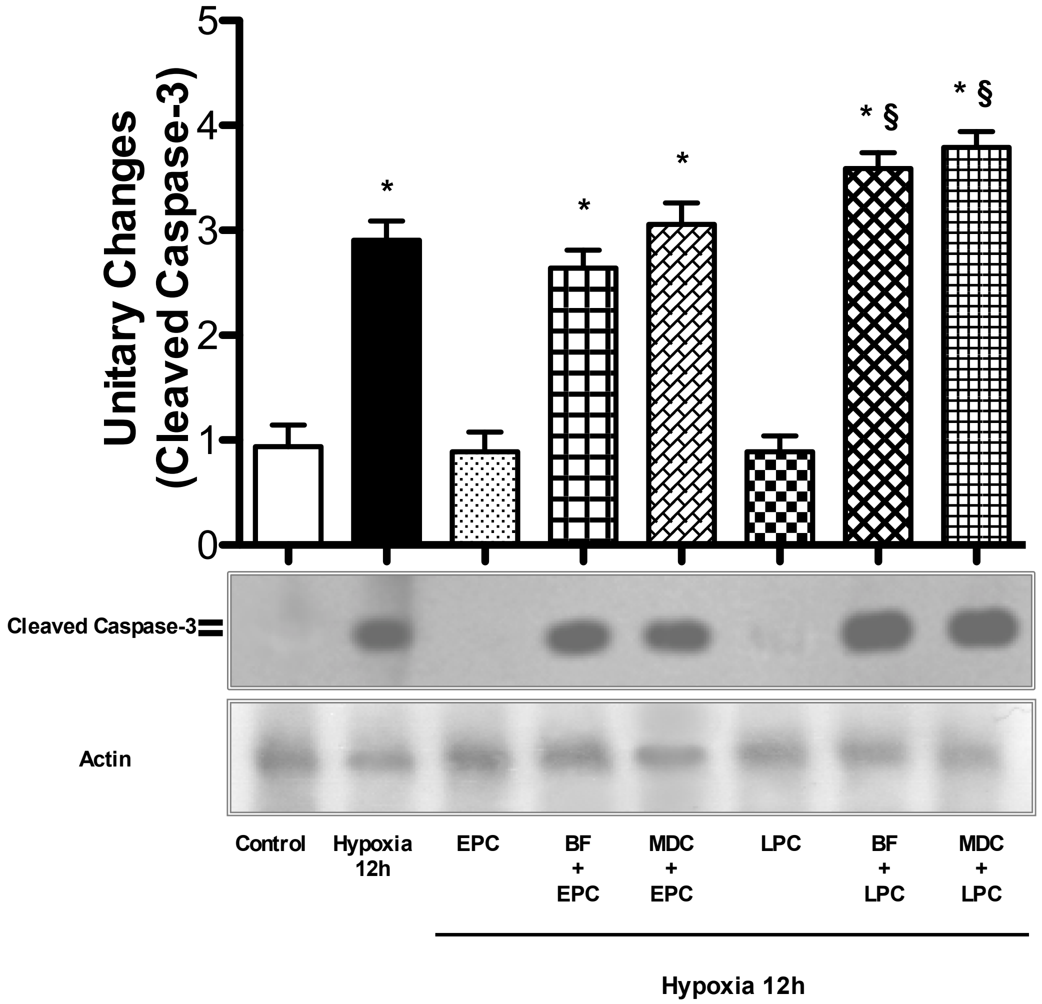

Figure 5. Cells were subjected to early and late preconditioning (EPC and LPC), in absence and in presence of the endocytosis inhibitor monodansylcadaverine (MDC) and of the recycling inhibitor bafilomycin (BF), as reported in the text. Caspase-3 cleavage was assessed. Bar graph represents densitometric analysis, mean±SEM, expressed as fold increase in caspase-3 cleavage over that in control cells, of four independent experiments.* $p<0.001$ vs control, EPC and LPC; § $p<0.001$ vs hypoxia 12 h, BF + EPC and MDC + EPC, by one-way ANOVA with a post hoc test of HSD.

Figure S6

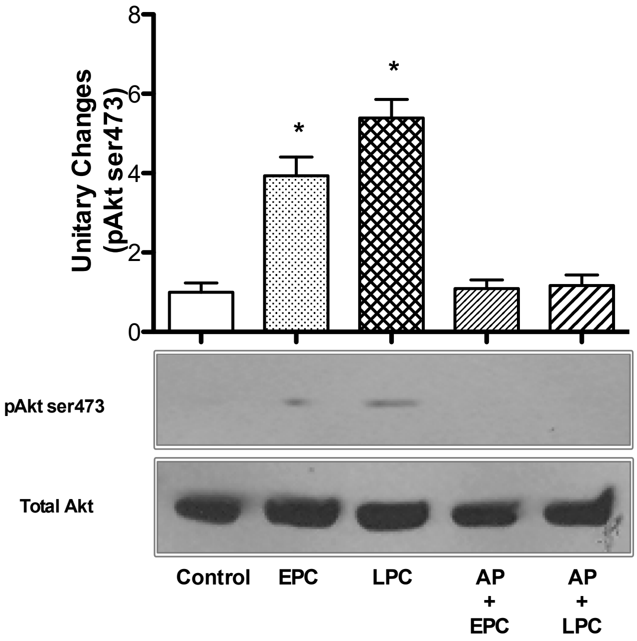

Figure 6. Cells were subjected to early and late preconditioning (EPC and LPC), in absence and in presence of aprotinin (AP), as reported in the text. Phosphorylation of Akt was assessed. Bar graph represents densitometric analysis, mean±SEM, expressed as fold increase in protein phosphorylation and expression over that in control cells, of three independent experiments.* p<0.001 vs control, by one-way ANOVA with a post hoc test of HSD.

Supplemental Tables

Table S1

Rate of apoptotic (Annexin V) and necrotic (Annexin V + Propidium Iodide) cell death following 12 hours of hypoxia preceded by stimulation with exogenous bradykinin (Bk 10^{-12} M and Bk 10^{-11} M) and by early and late preconditioning in the absence or presence of aprotinin (AP; selective tissue kallikrein inhibitor).

	**Annexin V**	**Annexin V + Propidium Iodide**
Control	5±2%	3±1%
Hypoxia 12 h	48±5%*	11±3%*
Bk 10^{-12} M + Hypoxia 12 h	27±5%*†	6±3%*†
EPC + Hypoxia 12 h	25±5%*†	6±2%*†
AP + EPC + Hypoxia 12 h	46±3%*	11±5%*
Bk 10^{-11} M + Hypoxia 12 h	26±2%*†	5±2%*†
LPC + Hypoxia 12 h	28±4%*†	8±2%*†
AP + LPC + Hypoxia 12 h	49±4%*	12±4%*

The rate of apoptotic and necrotic cell death was calculated by dividing the number of Annexin-V-positive/PI-negative cells and annexin-V-positive/PI-positive cells by the total number of nuclei detected with DAPI staining, respectively. The rate of apoptotic and necrotic cell death was calculated from four independent experiments. Bk: bradykinin, EPC: Early preconditioning, LPC: Late preconditioning.* $p<0.05$ vs control; † $p<0.05$ vs hypoxia 12 h, by a Chi-Square test.

Table S2

Rate of apoptotic (Annexin V) and necrotic (Annexin V + Propidium Iodide) cell death following 12 hours of hypoxia preceded by early and late preconditioning in the absence or presence of desArg10-HOE140, HOE140, transfection with short interfering-RNA for bradykinin receptor 1 and 2, bafilomycin and monodansylcadaverine.

	**Annexin V**	**Annexin V + Propidium Iodide**
Control	4±2%	2±1%
Hypoxia 12 h	49±3%*	10±3%*
EPC + Hypoxia 12 h	25±5%*†	4±2%*†
DesArg10-HOE140 + EPC + Hypoxia 12 h	27±5%*†	6±2%*†
siRNA-BKR1 + EPC + Hypoxia 12 h	28±4%*†	5±2%*†
HOE140 + EPC + Hypoxia 12 h	47±3%*	12±5%*
siRNA-BKR2 + EPC + Hypoxia 12 h	50±3%*	10±6%*
BF + EPC + Hypoxia 12 h	46±3%*	11±5%*
MDC + EPC + Hypoxia 12 h	47±2%*	11±2%*
LPC + Hypoxia 12 h	27±5%*†	7±1%*†
DesArg10-HOE140 + LPC + Hypoxia 12 h	45±4%*	13±4%*
siRNA-BKR1 + LPC + Hypoxia 12 h	46±2%*	14±4%*
HOE140 + LPC + Hypoxia 12 h	51±4%*	12±2%*
siRNA-BKR2 + LPC + Hypoxia 12 h	49±1%*	11±4%*
BF + LPC + Hypoxia 12 h	48±4%*	10±2%*
MDC + LPC + Hypoxia 12 h	49±4%*	12±4%*

The rate of apoptotic and necrotic cell death was calculated by dividing the number of Annexin-V-positive/PI-negative cells and annexin-V-positive/PI-positive cells by the total number of nuclei detected with DAPI staining, respectively. The rate of apoptotic and necrotic cell death was calculated from four independent experiments. EPC: Early preconditioning, LPC: Late preconditioning, siRNA-BKR1: short interfering-RNA for bradykinin receptor 1, siRNA-BKR2: short interfering-RNA for bradykinin receptor 2, BF: bafilomycin, MDC: monodansylcadaverine.* $p<0.05$ vs control; † $p<0.05$ vs hypoxia 12 h, by a Chi-Square test.

References

1. Condorelli G, Roncarati R, Ross J, Jr. et al. Heart-targeted overexpression of caspase3 in mice increases infarct size and depresses cardiac function. *Proceedings of the National Academy of Sciences of the United States of America* 2001; **98**: 9977-82.
